# Subcutaneous and continuous blood pressure monitoring in an ambulatory sheep by piezoelectric micromachined ultrasonic transducers

**DOI:** 10.1038/s41378-025-01019-w

**Published:** 2025-11-06

**Authors:** Yande Peng, Fan Xia, Zhichun Shao, Sedat Pala, Wei Yue, Hong Ding, Jin Xie, Liwei Lin

**Affiliations:** 1 Department of Mechanical Engineering, University of California, Berkeley, CA 94720 USA; 2 Department of Nanoengineering, University of California San Diego, La Jolla, CA 92092 USA; 3 The State Key Laboratory of Fluid Power and Mechatronic Systems, Zhejiang University, Hangzhou, Zhejiang 310027 China

**Keywords:** Engineering, Nanoscience and technology

## Abstract

This paper presents subcutaneous and continuous blood pressure (BP) monitoring using aluminum nitride (AlN) piezoelectric micromachined ultrasonic transducers (PMUTs) in an ambulatory sheep. A 37 $\times$ 45 PMUTs array with a footprint of 5 $\times$ 5 mm^2^ has been designed and fabricated as a prototype device. The deep reactive ion etching (DRIE) process to open the backside holes on the silicon substrate has been optimized to create active device diaphragms with a radius of 29 μm. The resulting PMUT unit has a measured resonant frequency of 6.5 MHz in water, an output acoustic pressure of 28 kPa at a distance of 10 mm, and a 6-dB bandwidth of about 33%. The BP monitoring scheme is validated through both in vitro and in vivo experiments to illustrate the correlation between the diameter of the blood vessel and pressure. Simulations indicate that possible issues in misalignment between the device and the blood vessel can result in a 60% reduction in signal strength with only 1 mm in misalignment. This highlights the advantage of subcutaneous implantation in maintaining a stable interface and consistent alignment for reliable long-term BP monitoring, in contrast to similar approaches via wearable system setups. The in vivo testing result shows BP wave fine features such as dicrotic notches and the averaged systolic/diastolic pressure errors are −1.2 $\pm$ 2.1 and −2.9$\pm$1.4 mmHg, respectively, which meets the clinical standard as calibrated by a gold-standard arterial line pressure sensor. As such, this system highlights the potential applications in silent, continuous, and highly accurate BP monitoring for hypertension patients using this implantable MEMS-based technology.

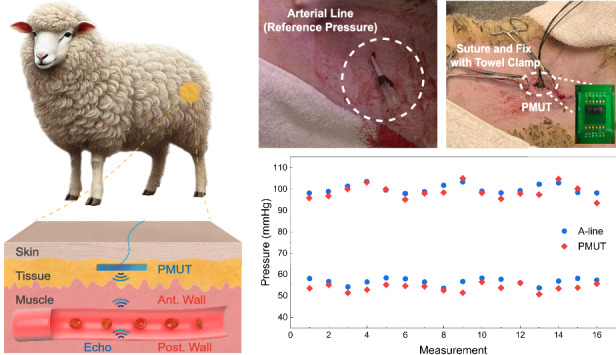

## Introduction

Hypertension, or high blood pressure (BP) is a leading risk factor for cardiovascular diseases, such as heart attacks, strokes, and heart failures and has been a major cause of medical expenses in the world^1,2^. For example, the United States of America devoted $131 billion in health costs to fight hypertension in 2022, with 685,875 associated deaths^3^. Statistically, more than 77.5% of hypertension adults with medications still don’t have good control over their BP^4^, which often results in excessive strain on the cardiovascular system to cause coronary artery disease^5^, heart failure^6^, and other complications. Research has demonstrated that an effective strategy to fight hypertension is intensive BP control through monitoring ambulatory BP regularly^7,8^. For example, 25% less cardiovascular events and 27% lower mortality rate have been reported if the systolic pressure can be reduced from 140 to 120 mmHg by monitoring BP regularly and adjusting medications accordingly^8,9^. However, conventional BP measurements based on the tonometry method require the inflation and deflation of the pressure cuff^10^, which severely disrupts normal activities and may cause allergic contact dermatitis for long-term usages^11,12^. As such, alternative approaches for long-term BP monitoring have been proposed by various schemes. One widely investigated method is to use the photoplethysmography (PPG) detector to measure blood volume changes to extract BP values^13,14^. However, PPG has limited penetration depth^15^, making it challenging to access arteries deep into the human body, such as the brachial artery (>10 mm) or central vasculature (~30 mm)^16^. In addition, PPG is highly susceptible to environmental interferences such as light, heat, and moisture, which affect measurement accuracy^17,18^. Ultrasound-based schemes have also been proposed for continuous BP monitoring with several reported wearable ultrasound patches, but they have issues with the interfaces between sensor and skin by using ultrasound gels^19–22^, which limit the monitoring duration for each operation^23^. Furthermore, misalignment presents a critical challenge^20^ in wearable systems as it can reduce the signal strength significantly to compromise accuracy. In order to address the challenges associated with interface variation and misalignment, implantable BP monitoring systems have been extensively investigated for clinical applications by various sensing mechanisms, including piezoresistive^24,25^, inductor-capacitor resonance^26^, capacitive^27^, and piezoelectric^28^ schemes. This work represents the first non-intrusive, implantable blood pressure monitoring based on ultrasound with good accuracy, as validated by in vivo experimental measurements. Most existing approaches^24,26,27^ apply intrusive implantations to place sensors inside the artery or cranium for direct pressure detections with the risk of inflammation such that post-operative care is required. On the other hand, implantable pressure sensors with non-intrusive implantation schemes^28,29^ often encounter issues due to the immune reaction with tissue growth over time at the device interface, which interferes the measurement accuracy. The proposed scheme is non-intrusive and it measures the time interval between ultrasound echoes from the two arterial walls to extract the artery diameter, which drastically reduces the immune reaction issue as the tissue growth impacts both ultrasound echo signals concurrently with minimum effect on the time interval characterization.

In this study, we introduce a non-intrusive subcutaneous BP monitoring system^30^ utilizing aluminum nitride (AlN) piezoelectric micromachined ultrasonic transducers (PMUTs)^31,32^ with a compact form factor of 5 × 5 mm^2^ fabricated by a CMOS-compatible process^33^. The operating principle is based on monitoring the diameter change of the blood vessel in response to pressure variations by extracting the pulse-echo ultrasound signals from PMUTs. We first conducted in vitro experiments to validate the correlation between artery wall diameter and applied blood pressure. Subsequent in vivo experiments in an ambulatory sheep showed the effective coupling between PMUTs and the biological environment to result in highly accurate BP measurements with <3 mmHg variations for both the systolic and diastolic BP as compared to the gold-standard measurement by an arterial line pressure sensor.

## Results and discussion

### Working principle and device design

**Fig. 1:**
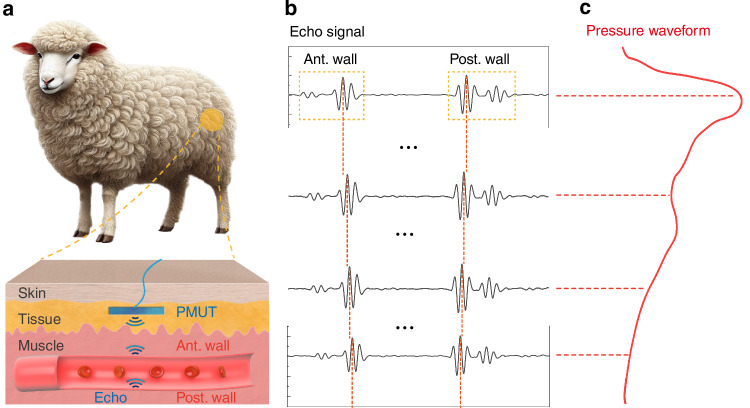
Illustration and principle of the subcutaneous and continuous BP monitoring by PMUTs in an ambulatory sheep. **a** The PMUT device is implanted subcutaneously near the sheep’s femoral artery to emit ultrasound and collect major echoes from interfaces at the artery’s anterior and posterior walls due to the large acoustic impedance mismatch between blood and arterial tissue. **b** Echoes from both walls are captured to estimate changes in the femoral artery’s diameter due to BP changes. **c** The extracted diameter changes are used to derive BP waveforms

The working principle is based on the real-time diameter monitoring of the blood artery using PMUTs shown in Fig. 1a. The device can be implanted subcutaneously above the target artery to emit ultrasonic waves and collect reflections from the artery-blood interfaces due to large differences in their acoustic impedances. The tissue and muscles are made of cells with very similar acoustic properties and generate negligible echoes. The time-of-flight (ToF) intervals between the two major echoes are used to extract the diameter of the artery, $D\left(t\right),$ based on the speed of sound in blood (1578 m/s) as shown in Fig. 1b. The diameter of the artery is related to the BP, $p\left(t\right),$ as^34^:

1 $$p\left(t\right)={p}_{d}\times {e}^{\beta \left(\frac{D\left(t\right)}{{D}_{d}}-1\right)}$$

where ${p}_{d}$, ${D}_{d}$ and $\beta$ are the diastolic pressure, diastolic arterial diameter, and vessel stiffness, respectively. By tracking changes in the blood arterial diameter $D(t)$, BP can be characterized in real-time (Fig. 1c).

The upper brachial artery is selected as target in this study for two main reasons: (1) its relatively large diameter (~4.5 mm)^35^ for more pronounced diameter changes during the cardiac cycles to enhance the signal-to-noise ratio; and (2) its similar height to the ventricle to minimize possible measurement errors from the height difference with respect to heart. Specifically, raising or lowering the monitoring location relative to the heart can introduce an error up to 20 mmHg by using the position of hand as an example^36^. The axial resolution of ultrasound signals determines the distinguishable distance between two features as:

2 $${{{Axial}}\;{{Resolution}}}=\frac{{{{spatial}}\;{{pulse}}\; {{length}}}}{2}=\frac{{{{number}}\; {{of}}\; {{pulses}}}}{2}\times \frac{c}{{{{frequency}}}}$$

where $c$ is the speed of sound. The thickness of the brachial artery can be correlated with various cardiovascular diseases^37^ such that the brachial artery thickness (~0.4 mm)^38^ is used as the critical axial resolution in this work. Since the ring-down length of PMUTs is around three cycles (Q ≈ 2 in water), as shown in Fig. S1, the minimum resonant frequency is calculated as 5.9 MHz. In practice, a high resonant frequency can enhance the resolution, but attenuation also increases as^39^:

3 $${p}_{{Rx}}\propto \frac{{V}_{{Tx}}}{R}\,\cdot\, {10}^{-\alpha \,\cdot\, f\,\cdot\, R}$$

where ${p}_{{Rx}}$ is the received pressure; ${V}_{{Tx}}$ is the transmitting voltage; $f$ represents the frequency of the PMUTs; $\alpha$ is the attenuation coefficient; and ${R}$ is the traveling distance. As such, the attenuation increases exponentially with both frequency and traveling distance. Considering these factors, a PMUT resonant frequency close to the minimum 5.9 MHz is chosen, and a circular-shaped PMUT design with a radius of 29 μm is calculated using finite element analysis in COMSOL based on our AlN PMUT structure and fabrication process (Fig. S2).

**Fig. 2:**
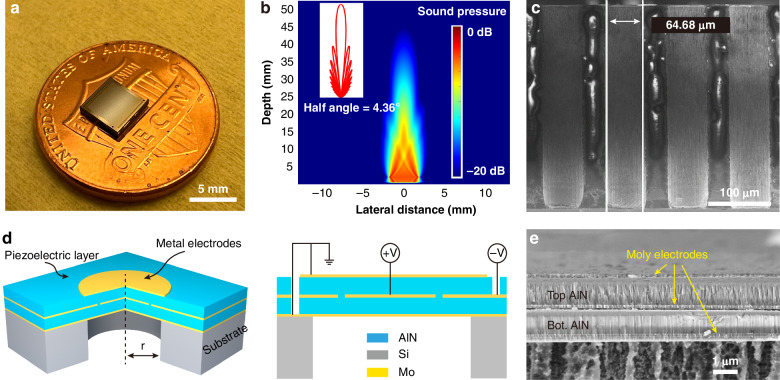
Device design and characterization. **a** Optical image showing the compact PMUT chip placed on top of a US one-cent coin. The chip is 5 × 5 mm^2^ and contains an array of 37 × 45 PMUTs. **b** Simulated acoustic emission field from the PMUT array with good beam directivity and penetration (40 mm). The theoretical half-pressure beamwidth is 4.36° as shown in the inset. **c** Cross-sectional SEM image of the backside trench with a good vertical profile using the optimized DRIE etching process. **d** 3D illustration and cross-sectional view of a bimorph dual-electrode PMUT. A segmented middle electrode with inner and outer regions driven by opposite polarities to enhance the output acoustic pressure. **e** Cross-section SEM image of the Mo/AlN/Mo/AlN/Mo stack with notable AlN columnar crystalline structure, highlighting the good crystallinity

Furthermore, an array design is adopted for higher echo signal strength and a 5 × 5 mm^2^ chip with an active 4 × 4 mm^2^ PMUT area is selected (Fig. 2a). The array has 37 × 45 individual PMUTs for 20 independent channels (17 channels with the base unit of 2 × 45 PMUTs, one rightmost channel and two leftmost channels with 1 × 45 PMUTs). This configuration generates a focused acoustic beam pattern at a penetration depth of about 40 mm, which is defined as the distance where the signal decays by ~20 dB (Fig. 2b) and a half-pressure beamwidth of 4.36°. The narrow beamwidth and adequate penetration depth ensure the acoustic energy is well-focused on the target artery to minimize reflections from surrounding tissues and organs at the edges. The theoretical directivity pattern $D(\theta )$ is calculated as^40^:

4 $$D\left(\theta \right)=\frac{\sin (\frac{N\,\cdot\, p\,\cdot\, k}{2}\sin \left(\theta \right))}{N\sin (\frac{k\,\cdot\, p}{2}\sin \left(\theta \right))}\,\cdot\, \frac{48{J}_{3}({kasin}\left(\theta \right))}{{({kasin}\left(\theta \right))}^{3}}$$

where $k=\frac{2\pi }{\lambda }$ is the wavenumber; *a* is the radius of the PMUT; $N$ is the number of channels, *p* is the pitch size (110 μm in this design); and ${J}_{3}$ denotes the Bessel functions of the first kind.

A bimorph dual-electrode design is adopted to produce high acoustic outputs^32,41^ using two active piezoelectric layers (Fig. 2d) with opposite-polarity electrodes in its inner and outer regions during operations and an inflection point at ~70% of the radius. The middle electrodes have an inner circular shape (covering 67% of the radius) and an outer ring shape. They are driven in opposite phases to match the polarity. This differential bimorph dual-electrode configuration theoretically results in a four-times increase in the bending moment and sensitivity. The fabrication process flow (Fig. S3) has been described in a previous work^41^, which begins with the deposition of a 200-nm-thick AlN layer by the AC sputtering scheme. This AlN layer serves as the seed layer for the crystal growth process along the c-axis for good piezoelectric properties, as well as the etching stop layer for the backside cavity definition. A 150-nm-thick molybdenum (Mo)/1-μm-thick AlN/150-nm-thick Mo film stack is deposited as the bottom electrode, bottom active piezoelectric layer, and middle electrode layer, respectively. The middle Mo electrode layer is then patterned and etched by an SF_6_ plasma etching process to form the inner and outer electrodes. Next, another 1-μm-thick top AlN layer and a 150-nm-thick Mo top electrode layer are deposited by the same AC sputtering process, followed by another SF_6_ plasma etch to define the top electrode. The AlN layer is then etched by a chlorine-based plasma process to open the bottom via and middle via for electrical contact pads to the corresponding electrodes. A 4-μm-thick oxide hard mask is deposited on the back of the wafer by a plasma-enhanced chemical vapor deposition (PECVD) process. The whole wafer is then attached to a dummy wafer for the backside deep reactive ion etching (DRIE) process to define the PMUT diaphragms. The DRIE process also acts as a dicing process to separate different chips by etching away their edges. Finally, the handle wafer is removed by dissolving the coupling grease in acetone. To achieve the small openings of the PMUT (29 μm in radius), the DRIE process is optimized in the PlasmaTherm Versaline ICP Etch system. Figure S4 shows the radius of etching profiles decreases as the etching depth increases with very rough surfaces and residual silicon pillars, probably due to the poor mass transport process for high-aspect-ratio features and the angular dispersion of ions in the DRIE process^42,43^. To solve this challenge, the wafer is grinded to 400 μm in thickness to reduce the aspect ratio and help the plasma dynamics. Furthermore, the pressure, applied power and bias voltage in the DRIE process are increased as the etching depth increases. Specifically, for every 100-μm-thick silicon etching process, the etching power is increased by ~10% to maintain a good etching profile. Figure 2c exhibits the cross-sectional SEM photo of a fabricated sample based on the modified recipe. A sidewall angle of 90.13° is achieved with a diaphragm radius of 29 μm (aspect ratio of 6.2 to 1) within about 10% variation of the designed value. Figure 2e displays the cross-section of a fabricated bimorph dual-electrode PMUTs, where the boundaries of each layer are well-defined. The columnar AlN structure observed from the SEM image indicates the good crystallinity for high-performance PMUTs.

### Device characterization

**Fig. 3:**
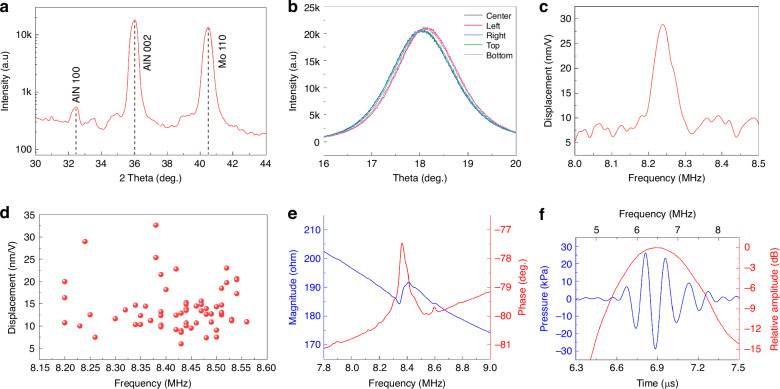
Material, electrical, mechanical, and acoustic characterization. **a** X-ray diffraction (XRD) characterization of the material stack showing distinct AlN and Mo peaks with high contrasts. **b** The rocking curve confirms the good uniformity of AlN thin film with an FWHM of around 1.56°. **c** Displacement vs frequency response of a single PMUT showing a sensitivity of 28 nm/V at a resonant frequency of 8.24 MHz in air. **d** Frequency and displacement distribution of 64 devices across the chip. **e** Impedance characterization of two central channels within the PMUT array in air. **f** Time- and frequency-analysis of the hydrophone signal

To further evaluate the deposited thin films, X-ray diffraction (XRD) is conducted. Figure 3a shows the locked couple scanning results, where the 100 and 002 diffraction peaks of the AlN thin film, and the 110 diffraction peak of the Mo electrode are observed, confirming their crystalline structures. The 002 orientation is the main contributor to the piezoelectricity. To evaluate both the crystallinity and uniformity of the thin film, rocking curve measurements are conducted at different locations across the wafer. As shown in Fig. 3b, the corresponding full-width at half-maximum (FWHM) values for the five representative points are 1.54°, 1.57°, 1.53°, 1.56°, and 1.56°. The small variation in FWHM values demonstrates excellent uniformity in the thin film for high crystalline quality.

A reflection digital holographic microscope (DHM, LynceeTec R-2100) is used to measure the mechanical resonances and displacements of PMUTs. Figure 3c shows the frequency response of a single PMUT, revealing a resonant peak at 8.24 MHz with an amplitude of 30 nm/V. The half-power bandwidth, determined by intersecting the curve at 70.7% of the peak displacement, is 0.05 MHz, corresponding to a quality factor (Q) of 164.8. The uniformity of the PMUT array is evaluated by measuring the resonant frequency and displacement amplitude for 64 devices on the same chip. Figure 3d shows that the center frequency is ~8.42 MHz with a frequency variation of 1% ($\sigma =0.092\,$ MHz). The displacement amplitude, with a mean of 13.95 nm/V, exhibits a variation of 36% ($\sigma =5.11$ nm/V), probably due to the non-uniform film stress and backside cavity etching process, which could be improved through enhanced process control.

The electrical impedance of PMUT is characterized by using Analog Discovery 2 by Digilent through its Impedance Analyzer function. A 4 × 45 PMUT array (corresponding to two channels) is tested, and Fig. 3e displays the impedance magnitude and phase vs. frequency information. The resonance f_r_ occurs at 8.34 MHz and the anti-resonance f_a_ at 8.41 MHz, with an impedance magnitude of 184.2 ohm at the resonance. The electromechanical coupling factor ${k}_{t}^{2}$ is extracted as 2.0% based on the f_r_ and f_a_. Finally, the acoustic performance of PMUT is characterized to evaluate its performance in water. During the measurement, an array with 8 × 45 neighboring elements is connected and excited using a 6 MHz rectangular pulse signal under an applied voltage of 30 V. A hydrophone (Onda HGL-0200) is positioned 10 mm away near the natural focal point to measure the output acoustic pressure. As shown in Fig. 3f, the PMUT has a resonant frequency of ~6.5 MHz when immersed in liquid and produces an output pressure of 28 kPa. The -6-dB bandwidth is as wide as 33% such that minor deviations in resonant frequency won’t significantly affect the operation. The resonant frequency in liquid (${f}_{{\mathrm{fluid}}}$) is lower than that in air (${f}_{{\mathrm{air}}}$) due to the additional medium loading, which can be expressed as^44,45^:

5 $${f}_{{{\mathrm{fluid}}}}=\frac{{f}_{{{{air}}}}}{\sqrt{1+0.67\cdot a\cdot {\rho }_{{{\mathrm{fluid}}}/\wedge }}}$$

where ${\rho }_{{fluid}}$ is the density of the liquid, $a$ is the radius of PMUT and $\Lambda$ is the PMUT mass per unit area. The added medium loading increases the damping of the PMUT, which results in improved coupling and a broader bandwidth.

### In vitro and in vivo tests

**Fig. 4:**
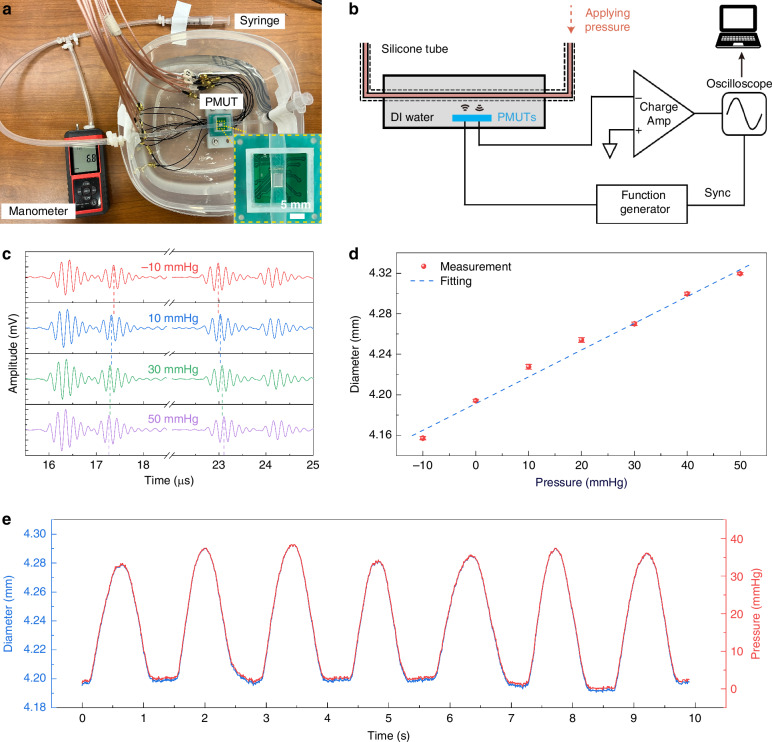
In vitro pressure monitoring by using PMUT. **a** Optical image of the in vitro experimental setup. The zoom-in image demonstrates the alignment of the tube above the PMUT. **b** System block diagram. **c** Static echo signals under different pressures. The interval between echoes increases as the applied pressure increases. **d** Diameter vs. pressure plot showing a good linear relationship. **e** Dynamic diameter/pressure waveform is obtained by continuously monitoring the tube diameter

To validate the proposed scheme for blood vessel diameter measurements, a dynamic in vitro experiment is conducted. Figure 4a illustrates the experimental setup, where the bimorph PMUT is mounted on a 3D-printed stage. A silicone tube with an inner diameter of 3 mm and an outer diameter of 4 mm (similar to a brachial artery) is used, which is aligned with the center of the PMUT array. A manometer is used to monitor the internal pressure and a syringe is used to adjust the applied pressure. A second syringe on the right side sealed the system to prevent leakage. Figure 4b outlines the data collection scheme. During the test, the PMUTs are driven at resonance by two cycles of rectangular pulse signals with an amplitude of 40 V_pp_ by a high voltage pulser (TX7516EVM, Texas Instruments) at a pulse repetition frequency of 4800 Hz. The received signals are amplified by two programmable amplifiers (VCA5807) and streamed to a laptop via an oscilloscope (PicoScope 5443D). To improve the signal quality, 24 signal groups per measurement cycle are averaged as shown in Fig. 4c under different pressures. It is observed that the time interval increases as the applied pressure increases, which implies that the tube diameter increases. The relationship between the tube diameter and applied pressure is calibrated in Fig. 4d with a linear relationship within the tested range as expected from Eq. (1). The small error bars indicate consistent and stable signal quality throughout measurements. The pressure inside the tube is modulated between 0 and 40 mmHg at a frequency of 1 Hz to emulate the heart rate and the dynamic diameter measurement is correlated with the applied pressure as shown in Fig. 4e. The reconstructed waveform reflects the applied pressure values and frequency, which confirms the feasibility by using PMUT for real-time blood vessel pressure monitoring.

**Fig. 5:**
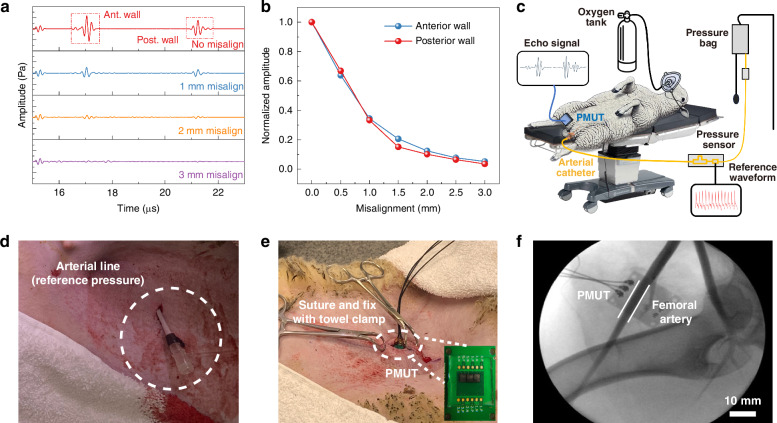
Possible misalignment effects and experimental setup for the PMUT chip in an adult sheep. **a** Simulation results showing ultrasound echoes under various misalignment conditions using k-Wave. **b** The normalized ultrasound echo amplitude reduction versus misalignment for the anterior (blue color) and posterior (red color) walls. **c** The implantation setup near the femoral artery of the sheep for the in vivo experiment. **d** The arterial line (gold standard) is used simultaneously to monitor the true BP values. **e** The PMUT was implanted near the femoral artery of the sheep during the in vivo experiment. **f** X-ray images verifying the proper relative position between the PMUT chip and the femoral artery

Ultrasound-based BP measurement is an indirect method, and the ultrasound echo signal strength depends on the relative positions of the PMUT device and the blood vessel. Misalignment between the sensor and the target artery can significantly compromise the measurement accuracy. Figure 5a illustrates the deterioration of ultrasound echo signals due to misalignment by MATLAB k-Wave toolbox simulations (details in the Methods section). It is observed that even a 1-mm misalignment can cause substantial signal degradation, and a 2-mm misalignment renders the signals almost indiscernible. Figure 5b further quantifies the impact, where the echo amplitude decreases by more than 60% under the condition of 1 mm in misalignment. For the case of a 2-mm misalignment, the signal strength reduces to ~10% of the original strength, which are consistent with experimental measurement results in Fig. S8. Clearly, the misalignment can affect sensing results in two areas. First, the acoustic path is altered, which affects the true artery diameter evaluations. Simulation results for the prototype setup show that a 1-mm misalignment introduces an error of ~10 ns, which corresponds to a ~10% error in the brachial artery diameter extension estimation (about 0.1 mm). Second, the reduction of the echo amplitude severely impacts the signal-to-noise ratio (SNR). Low SNR makes it challenging to differentiate the true signal from noise, resulting in unreliable BP estimates. These findings highlight the importance of good alignment or advanced mitigation techniques, such as beam steering, to ensure robust and accurate BP monitoring.

In this work, a subcutaneous implantation method is employed in an adult sheep with reliable alignment and stable interface coupling for potential long-term clinical usages. Adult sheep have similar physiological characteristics to those of a human, particularly in the vascular anatomy^46^. The implantation process is illustrated in Fig. 5c. Prior to the surgery, the adult sheep was fully anesthetized via intramuscular injection and fitted with an oxygen mask to ensure stable surgical conditions. The animal was positioned to maintain a stable posture, ensuring that its heart remained at a similar level to its hips. The furs over the left and right femoral arteries were shaved to facilitate clear implantation procedures. A gold-standard arterial catheter was inserted into the sheep’s left femoral artery and connected to a pressure transducer, which continuously recorded real-time blood pressure as the reference (Fig. 5d). On the right side, a 30-mm incision was made above the femoral artery using surgical scissors (Fig. S6). The PCB containing the PMUT sensor was then implanted subcutaneously, positioned above the center of the femoral artery, and adjusted for maximal echo signal amplitudes. Afterwards, the position of the PCB was secured with sutures and towel clamps to simulate a stable, real-world implantation scenario (Fig. 5e). To ensure intimate coupling between the PMUT and surrounding tissue, a saline wash bottle was used to flush the implantation site, effectively eliminating possible air bubbles before suturing. Following the implantation process, fluoroscopy was performed to confirm the relative position between the PMUT and the femoral artery. The PMUTs array used in the animal test is shown in the inset of Fig. 5e. A unimorph AlN PMUT with similar performance (Fig. S5 and Table S2) was used here with two transmitter (T_x_) chips on both sides and a receiver (R_x_) chip in the middle. To protect the bonding wires from mechanical damage, a layer of polydimethylsiloxane (PDMS) with a thickness of 4 mm was coated onto the array. PDMS is a biocompatible, easy-to-process, and transparent polymer with good chemical stability and is widely used in microfluidics and biomedical applications. Fluoroscopy (Fig. 5f) confirmed placement of the PMUT chip above the femoral artery, which was measured to be ~5 mm in diameter. Other surrounding structures, including veins and bones, are sufficiently distant to avoid interference. Due to resource constraints, the preliminary implantation study was limited to a single sheep. On the other hand, the long-term safety of implantable medical devices^47^ has been well established through decades of clinical use in systems such as cardiac pacemakers, cochlear implants, and implantable intraocular pressure sensors. A key consideration is to effectively isolate electronics from the surrounding biological environment. In this work, we utilize proven medical-grade polymers such as silicone rubber and polyurethane^48^, which are commonly used in implantable devices, and these materials also possess favorable acoustic properties as coupling layers in an ultrasound-based sensing system. Furthermore, materials of our PMUT device—aluminum nitride (AlN), molybdenum (Mo), silicon dioxide, and crystalline silicon—are widely recognized for their biocompatibility in various approved biomedical device^49–51^. Therefore, with an appropriate packaging strategy, the proposed system should be regarded as a safe implantable device.

**Fig. 6:**
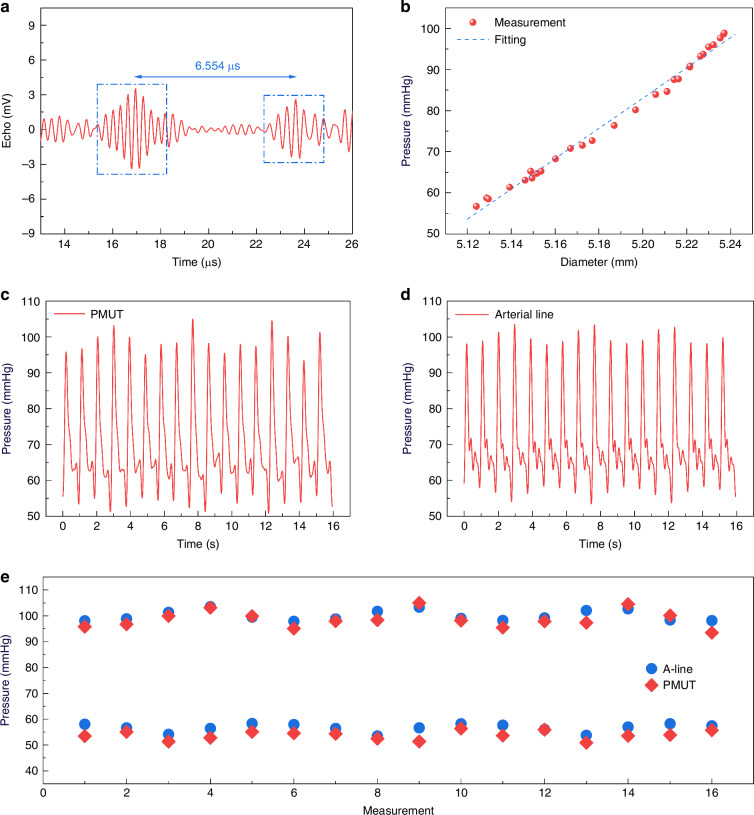
In vivo experimental results. **a** An example of echo signals collected during the in vivo experiment. **b** Correlation between the arterial pressure and the diameter shown with a fitted blue curve. BP waveform obtained by using the **c** PMUT and **d** pressure sensor in the arterial line. **e** Comparison of the systolic and diastolic pressure values to demonstrate the good accuracy

Figure 6a displays an example of the echo signal obtained during the animal test after a bandpass filtering process using MATLAB. There are several echoes due to the heterogeneous components in the sheep body and the strongest signals are from the artery/blood interfaces due to the significant acoustic impedance mismatch (Table S3). In this case, the anterior wall signal occurs at 16.03 μs, corresponding to a depth of 12.7 mm using the average speed of sound in typical sheep muscles at 1588 m/s^52^. The posterior wall signal is detected 6.55 μs later, indicating a wall-to-wall diameter of 5.17 mm, and it matches well with the observation under fluoroscopy. The PMUT sensor is calibrated by correlating the measured artery diameters to the BP values by the arterial line pressure sensor as shown in Fig. 6b with a good linear relationship with diameters ranging from 5.12 to 5.24 mm corresponding to BP from 56 to 99 mmHg.

The dynamic in vivo femoral diameter waveform is then converted to a BP waveform for a period of 16 s as shown in Fig. 6c using the BP values from the pressure sensor on the arterial line in Fig. 6d. Results show that the PMUT device also captures key cardiovascular features, such as the dicrotic notch, caused by a transient increase in aortic pressure upon the closure of the aortic valve^53^. Figure 6e compares the PMUT-based measurements to the arterial line pressure sensor readings with good agreement. The systolic and diastolic pressures measured by PMUTs differ from the arterial line by an average of −1.2 mmHg and −2.9 mmHg, respectively. Except for one outlier in the diastolic pressure, all measurements fall within a 5-mmHg range, which meets the standard of sphygmomanometers^54^. This highlights the feasibility of PMUT-based monitoring for accurate, continuous BP measurements. Compared to other reported implantable blood pressure monitoring systems (Table 1), our PMUT-based sensor demonstrates superior accuracy among non-intrusive methods and rivals the performance of some state-of-the-art intrusive blood pressure monitoring approaches. One significant advantage of using ultrasound for BP monitoring is its inherent resistance to interface and medium changes (e.g., immune reactions or tissue growth) over time. This stability arises because the key parameter for BP estimation—the echo interval between the two arterial walls—is primarily influenced by blood properties and alignment (can be maintained through suturing during the surgical), rather than the characteristics of the intermediate tissue. In contrast, methods that directly measure pressure are highly susceptible to pressure wave propagation variations, which are sensitive to interface materials and layer thickness^20^. Further experimental validation of our approach will be conducted in future work.

**Table 1:** Summary of implantable BP monitor systems

Method	Accuracy (mmHg)	Long-term Use	Intrusive^*^
Piezoresistive #1^24^	$\pm$3	No (biodegradable)	Yes
LC Resonance^26^	$\pm$8	Yes	Yes
SAW Resonator^57^	$> \pm$10	Yes	Yes
Piezoresistive #2^25^	$\pm$10	Yes	Yes
Piezoelectric #1^29^	$\pm$8	No	No
Capacitive^27^	$\pm$3	Yes	Yes
Piezoelectric #2^28^	$\pm$5	Yes	No
Ultrasound (this work)	$\pm$4.6	Yes	No

*Intrusive means the surgery requires implanting the sensors inside the artery or the cranium

## Conclusions

This work has demonstrated continuous and subcutaneous BP monitoring based on AlN PMUTs by extracting the pressure information from measured diameter changes of the artery vessel. A 5 mm by 5 mm chip comprising 37 × 45 bimorph dual-electrode PMUTs with a resonant frequency of 6.5 MHz in water has been used in the prototype setup experiment. The fabrication process has included an optimized DRIE recipe to construct features such as high-aspect-ratio holes. The morphological and crystalline analysis of fabricated films confirm good AlN quality (FWHM <1.6°) and fabricated PMUT devices show a resonant frequency around 8.42 MHz in air with 1% variations and a displacement of 13.95 nm/V with 36% variations for the diaphragms of 29 μm in radius. Hydrophone characterization confirms the bandwidth of 33% and output acoustic pressure of 28 kPa about 10 mm away at the natural focus. Real-time diameter monitoring based on a silicone tube with a similar geometry to the blood vessel has been successfully demonstrated with the real-time diameter change waveform measured by PMUTs and restored for in vitro experiments. The in vivo animal test has been conducted on an ambulatory sheep, and results have demonstrated subcutaneous and continuous measurements of BP with an averaged diastolic and systolic pressure error of −1.2$\pm$2.1 mmHg and −2.9$\pm$1.4 mmHg, respectively. Two possible directions are envisioned for future work. First, advanced schemes such as the beamforming technique should be developed to address the possible in-use misalignment issues. Specifically, the transmission beamforming scheme can be applied to locate and focus the ultrasound on the right spot to compensate potential location shifts due to daily activities. Second, the continuous monitoring scheme to prevent hypertension-related complications could present an exciting opportunity to investigate BP-linked chronic diseases, and the potential data-driven approach could advance preventative healthcare as a new direction for biomedical research.

## Materials and methods

### Simulation method

Finite element analysis (FEA) is employed to evaluate the resonant frequency of the PMUT device using the COMSOL® software (COMSOL Multiphysics® v. 6.0, COMSOL AB, Sweden). The simulation incorporates three interfaces: Solid Mechanics, Electrostatics, and Pressure Acoustics. A 2D axisymmetric model is adopted to leverage the symmetry of the device, and the resonant frequency is determined through the frequency domain analysis. The edge of the diaphragm is set as a fixed boundary to represent clamped conditions, and a perfectly matched layer (PML) is applied to the boundary of the air medium to eliminate unwanted interferences. Beam pattern and misalignment evaluations are conducted using MATLAB’s k-Wave toolbox. The simulation domain consists of a 2D space, ~26 × 26 mm^2^, with layers of skin, fat, muscle, blood vessels, and blood. Misalignment is defined as the horizontal distance between the centers of the PMUT array and the artery. Detailed simulation schemes and material properties are provided in Fig. S6 and Table S3. The grid size is set to ~1/10 of the wavelength (24 μm) to ensure adequate resolution. These parameters define a simulation space capable of supporting a maximum frequency of 30 MHz, with a Courant–Friedrichs–Lewy (CFL) number of 0.3 and a grid resolution of 102 × 1024 points, including a perfectly matched layer (PML) of 40 grid points.

### Acoustic interface

In order to protect the bonding wires from damage and insulate the PMUT from the biomedical medium during the in vivo test, a layer of polydimethylsiloxane (PDMS, Sylgard 184, Dow Corning) with a thickness of ~4 mm is coated onto the PMUT array. The Sylgard 184 PDMS is supplied as a two-part liquid component kit, a pre-polymer precursor (part A) and a cross-linking curing agent (part B). In this study, the two parts are mixed with a weight ratio of 10:1 and thoroughly stirred to ensure homogeneity to prevent curing inconsistencies. After mixing, the PDMS is degassed in a vacuum chamber to remove trapped air bubbles, which could otherwise create defects and block ultrasound waves. The degassed mixture is then deposited onto the substrate and cured in an oven at 60 °C for 1 h to initiate cross-linking between polymer chains. PDMS is biocompatible, easy-to-process, and transparent with good chemical stability for microfluidics and biomedical applications^55,56^. Additionally, its low Young’s modulus allows for minimal mechanical interference with delicate biological tissues as a protective coating for sensitive electronic components in wearable or implantable devices.

### In vivo animal implantation

This detailed implantation protocol is designed to ensure optimal acoustic coupling, stable signal acquisition, and long-term functionality of the PMUT-based blood pressure monitoring system, while maintaining animal welfare throughout the procedure. Prior to the surgery, the adult sheep was fully anesthetized via intramuscular injection using a combination of xylazine and ketamine to induce deep sedation and muscle relaxation. To maintain adequate anesthesia, additional doses were administered as needed during the procedure. The animal was fitted with an oxygen mask to ensure proper oxygenation throughout the surgery. The sheep was carefully positioned to maintain a stable posture, ensuring that its heart remained at the same level as its hips to minimize hydrostatic pressure differences that could influence blood pressure measurements. During the entire procedure, the animal’s anesthetic state was continuously monitored by assessing jaw tension, heart rate, blood pressure, end-tidal CO_2_, and oxygen saturation. These parameters were tracked to ensure stable anesthesia and physiological stability. A gold-standard arterial catheter was inserted into the left femoral artery and connected to a pressure transducer to continuously record real-time blood pressure, serving as a reference measurement. To maintain catheter patency and prevent clot formation, heparinized saline was periodically flushed through the line. On the right side, a 30-mm longitudinal incision was made directly above the femoral artery using surgical scissors, while carefully avoiding major blood vessels and nerves. The subcutaneous tissue was gently dissected to create an implantation pocket. The PCB containing the PMUT sensor was then implanted subcutaneously, positioned precisely above the center of the femoral artery, and adjusted to maximize the echo signal amplitude. After achieving the optimal positioning, the PCB was secured with non-absorbable sutures at multiple anchoring points to maintain stable positioning. To further immobilize the implanted PCB, towel clamps were used to anchor the surrounding tissue, simulating a stable, real-world implantation scenario. To ensure intimate coupling between the PMUT and the surrounding tissue, the implantation site was thoroughly flushed with sterile saline using a wash bottle to eliminate any air bubbles, which could compromise acoustic coupling. After confirming the absence of air pockets, the incision was closed with interrupted sutures, and the area was covered with a sterile dressing. Post-surgery, the sheep was continuously monitored for vital signs and observed for post-operative stability, including checks for hematoma, edema, or other complications at the implant site. The animal was carefully monitored during the recovery phase to ensure that the implantation did not cause any adverse health effects.

## Supplementary information

Supplemental Material
