# Supplementary material for: Subcutaneous and continuous blood pressure monitoring in an ambulatory sheep by piezoelectric micromachined ultrasonic transducers
Source: Microsyst Nanoeng. 2025 Nov 6;11:207. doi: 10.1038/s41378-025-01019-w (PMC12592419; doi:10.1038/s41378-025-01019-w)
Supplement: Supplementary file 1 — Supplemental Material [file 41378_2025_1019_MOESM1_ESM.docx]

Subcutaneous and continuous blood pressure monitoring in an ambulatory sheep by piezoelectric micromachined ultrasonic transducers

Yande Peng^1^, Fan Xia^1^, Zhichun Shao^1^, Sedat Pala^1^, Wei Yue^1^, Hong Ding^2^, Jin Xie^3^, Liwei Lin^1^

1 Department of Mechanical Engineering, University of California, Berkeley, CA 94720, USA

2 Department of Nanoengineering, University of California San Diego, La Jolla, CA 92092, USA

3 The State Key Laboratory of Fluid Power and Mechatronic Systems, Zhejiang University,

Hangzhou, Zhejiang 310027, China

Corresponding author: [lwlin@berekely.edu](mailto:lwlin@berekely.edu)

**Supplementary Information**

**Figure S1. Typical echo signal and corresponding axial resolution.**

(a) An example of the echo signal from simulation results, the left two echoes are from the two sides of the anterior wall and the right two echoes are from the posterior wall. (c) Illustration of the spatial resolution length (SPL) and axial resolution.


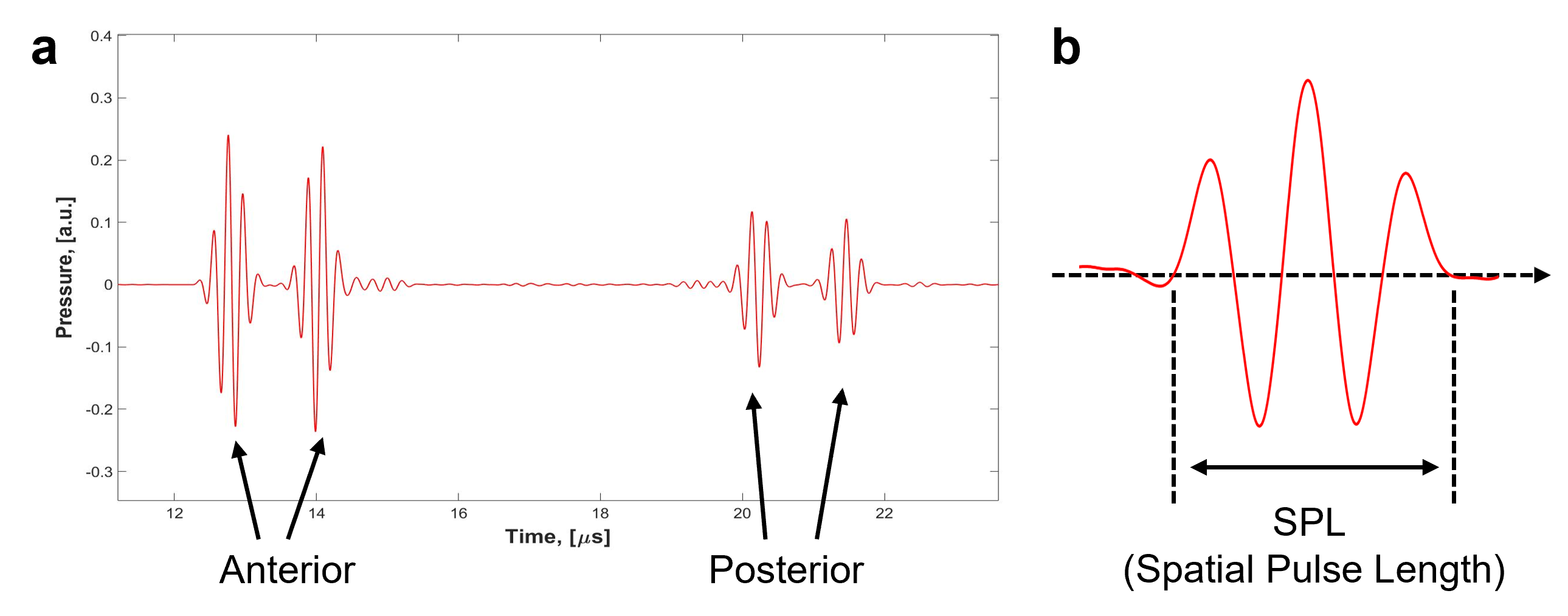


**Figure S2. PMUT mode shape and resonance frequency.**

(a) Fundamental mode shape of PMUTs from COMSOL simulation. (b) Resonance frequency in water changes with radius.


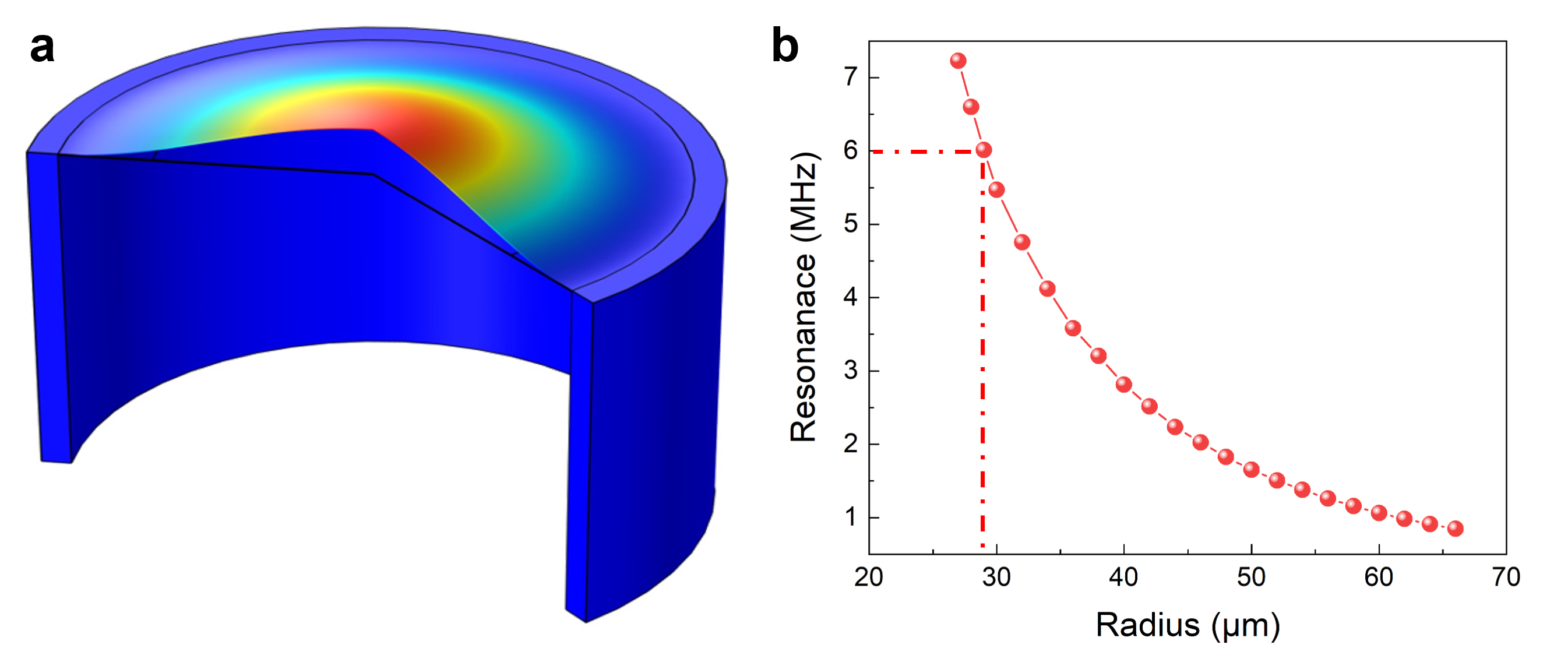


**Figure S3. The PMUT fabrication process.**

(a) sputtering of seed AlN/Mo/AlN/Mo stack, the seed AlN is used to improve the quality of the AlN above; (b) middle Mo electrodes patterning; (c) top AlN/Mo sputtering and electrodes patterning; (d) patterning of middle via; (e) bottom via patterning; (f) DRIE backside etch.


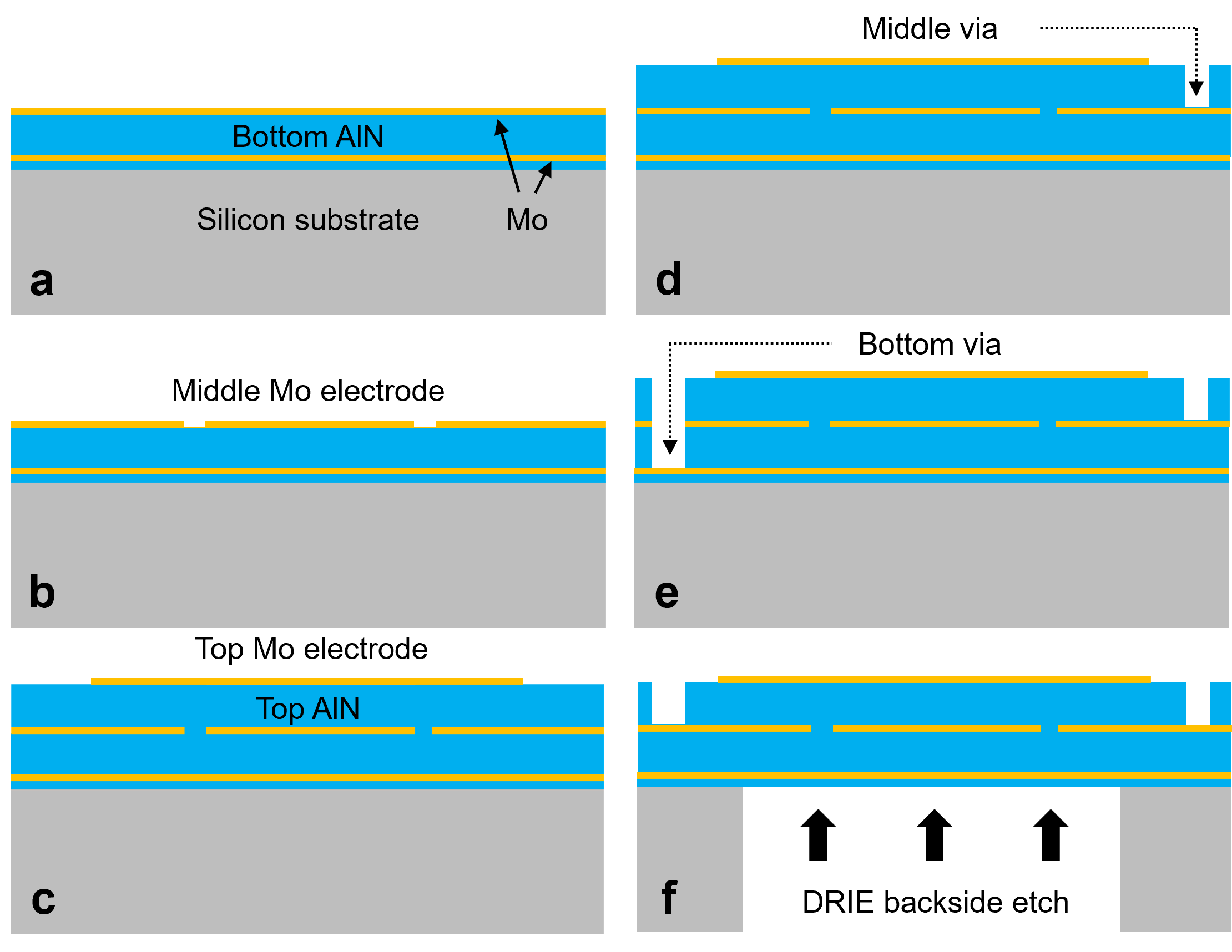


The process begins with the deposition of a 200-nm AlN layer using AC sputtering. This layer acts as the seed layer for later crystal growth, promoting the AlN layer on it to grow along the c-axis orientation for good piezoelectric properties^1,2^. Additionally, this AlN layer shows inertness in SF_6_-based plasma chemistries, which is necessary in the backside etch and serves as an excellent stop layer, improving yield. We then deposit a 150-nm Molybdenum (Mo)/1-μm AlN/150-nm Mo film stack as the bottom electrode, bottom active piezoelectric, and middle electrode layers, respectively. The middle Mo electrodes are then patterned by SF6 plasma etch to form the inner and outer electrodes for differential driving and receiving. Next, another 1-μm thick top AlN layer and 150-nm thick Mo top electrode layer are deposited through the same AC sputtering process, followed by another SF_6_ plasma etch to define the top electrode regions. The AlN is then etched by chlorine-based plasma to open the bottom via and middle via to obtain electrical connections to the corresponding electrode. A 4-μm oxide hard mask is deposited on the back of the wafer through plasma-enhanced chemical vapor deposition (PECVD). The whole wafer is then attached to a dummy wafer for the backside DRIE to define the diaphragm. The DRIE process also acts as dicing to separate the different dies directly by etching away the edges. This method allows us to skip the traditional dicing process and reduces the clearance needed for mechanical or laser dicing, making better use of the wafer area. Finally, we remove the handle wafer by dissolving the coupling grease in acetone and obtain the single chips.

**Figure S4. Scanning electron microscope (SEM) images of the undesired etch profile of PMUT**

(a) An overview of the entire etched trench. (b) A zoom-in image of the bottom part which is narrowed and rough. (c) A zoom-in image of the top part, which is relatively smooth and uniform. (d) A zoom-in image of the etched region near the diaphragm, which shows pillar-like profiles at the edge of the trench.


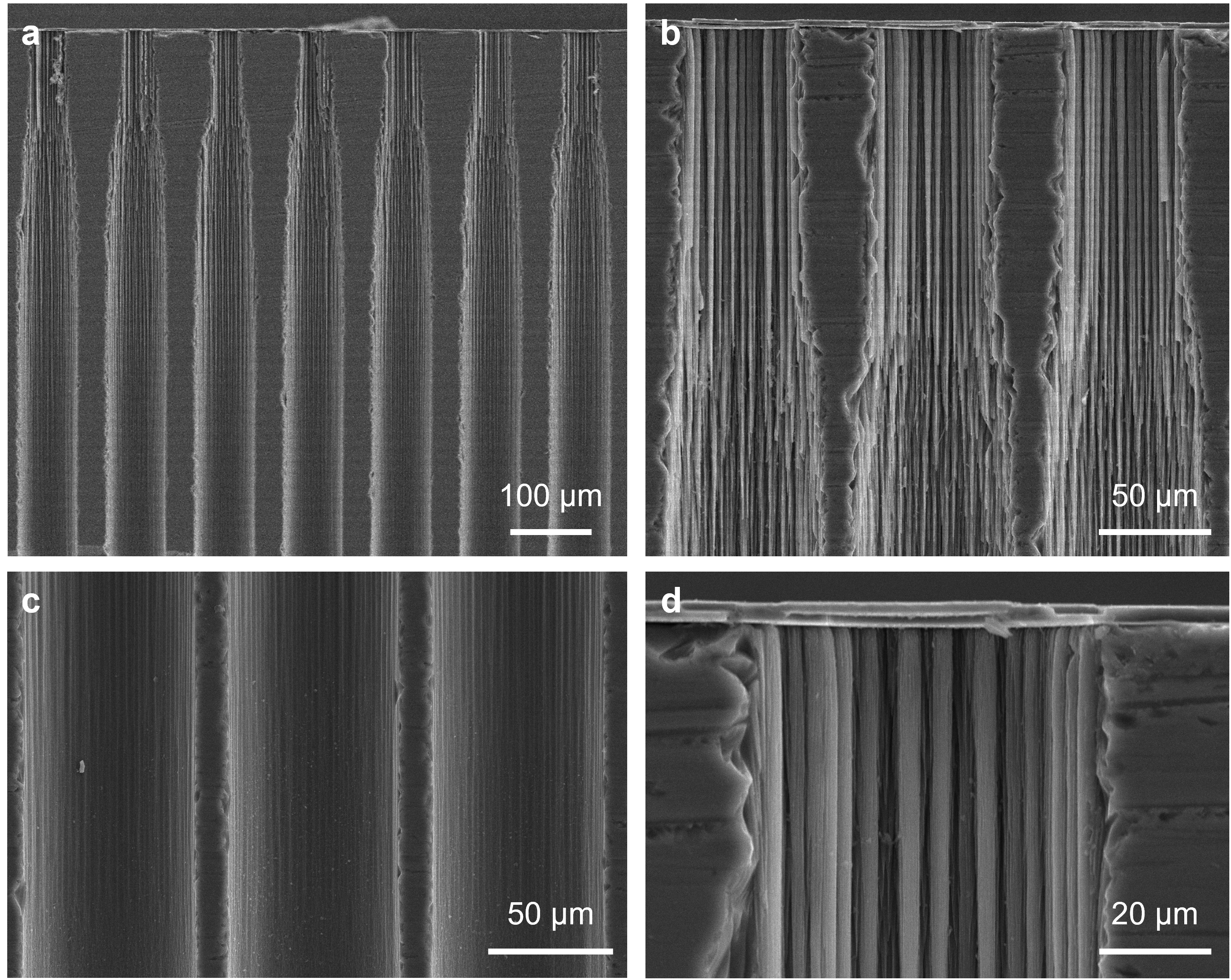


The fabrication of high frequency PMUTs through DRIE process posed significant challenges due to the high aspect ratio backside holes, leading to the failure of the fabrication attempt. **Figure S4** depicts the scanning electron microscopy (SEM) of the etched PMUT cross-section using the previous recipe, and it is evident that the radius of the etch profile decreases as it approaches the diaphragm (**Figure S4a** and **S4b**). The bottom part of the trench (closer to the diaphragm) profile is rough, and residual pillars remain at the profile's edge (**Figure S4d**). This unsatisfactory etch profile significantly impacts the PMUT's performance. Firstly, it changes the geometry of the diaphragm, and the rough edge profile changes the mechanical boundary conditions as well. Secondly, the changed electrode coverage weakens the PMUT's performance.

**Figure S5. Echo signals of two version of PMUTs from similar measurement**

(a) Echo collected by unimorph PMUT with an amplitude of 2.12 mV. (b) Echo collected by bimorph PMUT with an amplitude of 1.01 mV.


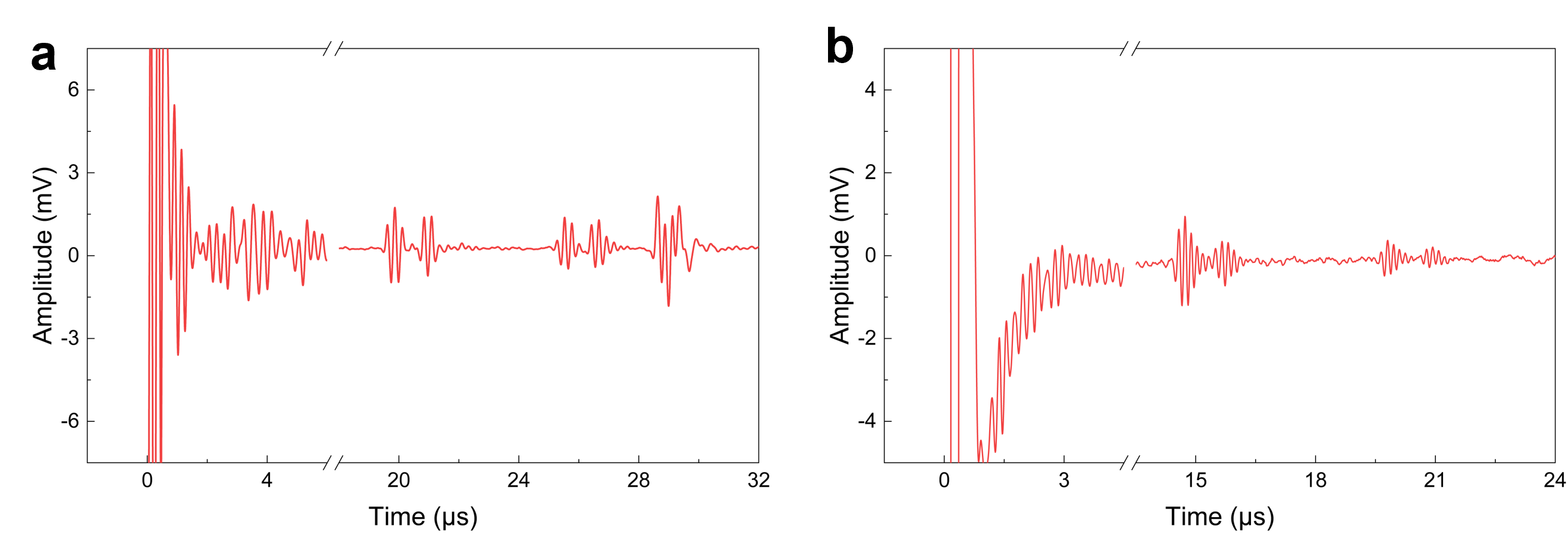


The pulse-echo measurement performance was compared between two versions of PMUTs. In the unimorph PMUT system, the two edge arrays were used for excitation (each array with 23 × 26 PMUTs), while the middle array served as the receiver. In the bimorph PMUT system, half of the array (20 × 45 PMUTs) is used as transmission and only two central channels (4 × 45 PMUTs) were used for receiving. The lower signal amplitude observed with the bimorph PMUT is primarily attributed to the smaller receiving sensor area, as a charge amplifier was employed. By utilizing additional channels or incorporating all channels via a transmit/receive (T/R) switch, the bimorph PMUT system could achieve superior signal performance compared to the unimorph PMUT arrays used in the animal study.

**Figure S6. Photo of the surgical procedure.**

(a) Optical images showing the geometry of the left femoral artery in an adult sheep. (b) PMUT sensor implantation during the surgery.

**
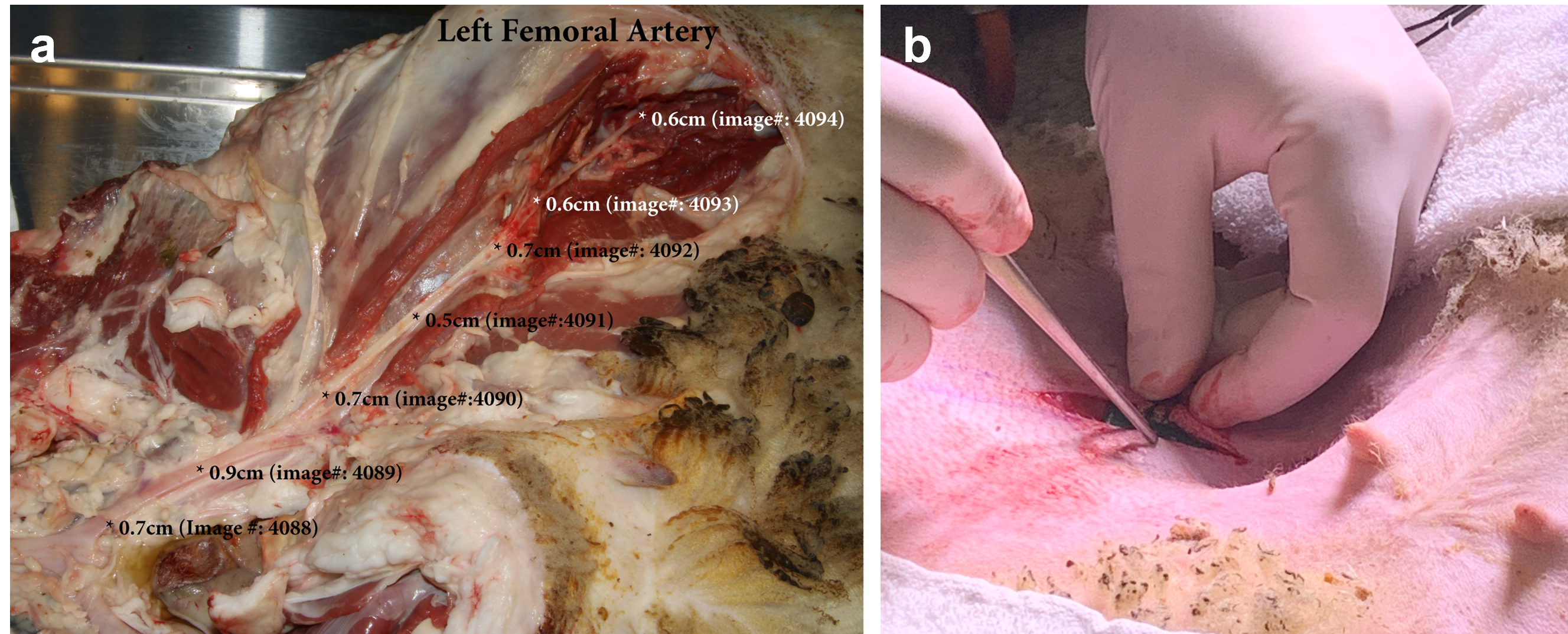
**

**Figure S7. K-Wave Simulation Setup**

**
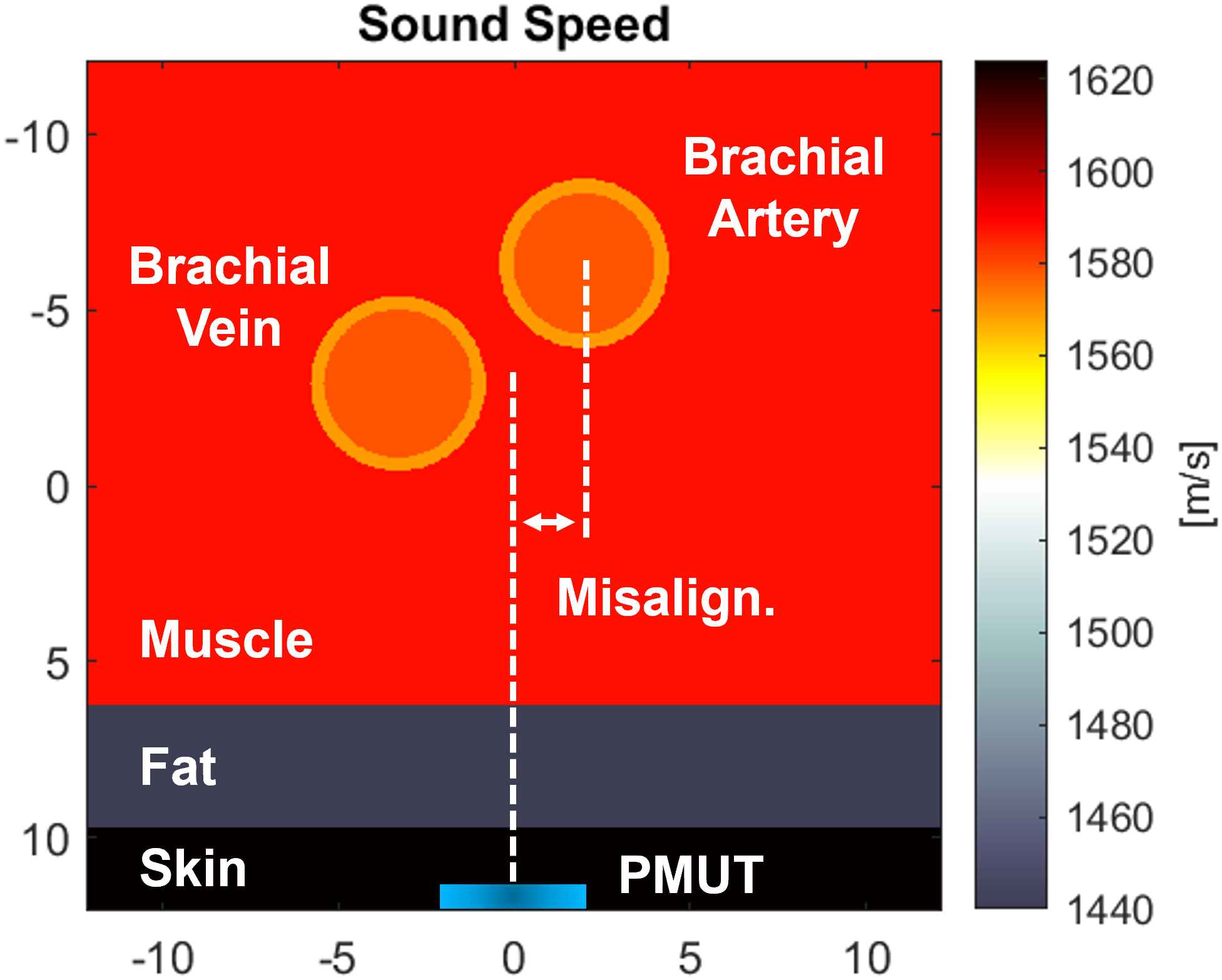
**

**Figure S8.** **Experimental results for various lateral misalignments (**$\boldsymbol{\Delta}$**) with received echo signals.**





**Table S1. Correlation between DRIE process parameters and the etch profiles**^3,4^**. ↑ indicates a positive correlation, ↓ indicates a negative correlation, ↔ indicates no effect or negligible effects.**

|  | Bias power | Etching pressure | SF_6_ flow rate | C_4_F_8_ flow rate | Etch:Dep time ratio |
| --- | --- | --- | --- | --- | --- |
| Etching rate | $\uparrow$ | $\uparrow$ | $\uparrow$ | $\downarrow\leftrightarrow$ | $\uparrow$ |
| Sidewall angle | $\downarrow$ | $\downarrow$ | $\downarrow$ | $\leftrightarrow$ | $\downarrow$ |
| Sidewall damage | $\uparrow$ | $\uparrow$ | $\uparrow$ | $\downarrow$ | $\uparrow$ |

**Table S2. Comparison between the two version of PMUTs**

| Excitation | Unimorph PMUT | Bimorph PMUT |
| --- | --- | --- |
| 30 V_pp_ | 12.5 kPa | 15.4 kPa |
| 60 V_pp_ | 22.8 kPa | 28 kPa |

Two versions of PMUTs were characterized using a hydrophone (Onda HGL200) under varying excitation voltages. For the unimorph PMUT, shown in **Figure 5b**, the pressure measurements were conducted by exciting the PMUT arrays on both sides, each consist of 23 × 26 PMUT elements. The bimorph PMUT, depicted in **Figure 2a**, consist of 37 × 45 PMUT elements of the array (20 × 45 PMUTs). For pressure characterization, half of the bimorph array (20 × 45 elements) was excited. The results show with only half of the area, the bimorph PMUT generate slightly higher pressure compared to that of unimorph PMUTs, which correspond with previous reports.

**Table S3. Medium properties in the simulation**^5^

|  | Thickness (mm) | Density (kg/m^3^) | Speed of sound (m/s) | Attenuation coefficient (dB/cm @ 1 MHz) |
| --- | --- | --- | --- | --- |
| Skin | 1.3 | 1109 | 1624 | 1.84 |
| Fat | 3.5 | 911 | 1440 | 0.38 |
| Muscle | \ | 1090 | 1588 | 0.62 |
| Blood vessel | 0.37 | 1102 | 1569 | 0.61 |
| Blood | \ | 1050 | 1578 | 0.21 |

**References**

1. Ababneh, A., Schmid, U., Hernando, J., Sánchez-Rojas, J. L. & Seidel, H. The influence of sputter deposition parameters on piezoelectric and mechanical properties of AlN thin films. *Materials Science and Engineering: B* **172**, 253–258 (2010).

2. Ababneh, A. *et al.* C-axis orientation and piezoelectric coefficients of AlN thin films sputter-deposited on titanium bottom electrodes. *Appl Surf Sci* **259**, 59–65 (2012).

3. Rangelow, I. W. Critical tasks in high aspect ratio silicon dry etching for microelectromechanical systems. *Journal of Vacuum Science & Technology A: Vacuum, Surfaces, and Films* **21**, 1550–1562 (2003).

4. Xu, T., Tao, Z., Li, H., Tan, X. & Li, H. Effects of deep reactive ion etching parameters on etching rate and surface morphology in extremely deep silicon etch process with high aspect ratio. *Advances in Mechanical Engineering* **9**, (2017).

5. The Foundation for Research on Information Technologies in Society (IT’IS). https://itis.swiss/virtual-population/tissue-properties/database.
